# Unmasking Vitamin B12 Deficiency Misdiagnosed as Myelodysplastic Syndrome

**DOI:** 10.1155/2024/3258227

**Published:** 2024-12-02

**Authors:** Maria Jamil, Zeinab Nasser, Dawood Jamil, Jawad Z. Sheqwara

**Affiliations:** ^1^Department of Internal Medicine, Henry Ford Health System, Detroit, Michigan, USA; ^2^Department of Hematology-Oncology, Henry Ford Health System, Detroit, Michigan, USA

**Keywords:** MDS, myelodysplastic syndrome, pancytopenia, pernicious anemia, vitamin B12 deficiency

## Abstract

**Background:** Pancytopenia is characterized by a decrease in all three types of blood cells. Instead of being a standalone disease, it acts as a common outcome resulting from various factors, including infections, autoimmune disorders, genetic issues, nutritional deficiencies, and malignancies. Pinpointing the root cause of pancytopenia poses a challenge but is essential for devising an effective treatment plan and predicting the likely prognosis. Vitamin B12 deficiency is a common cause of megaloblastic anemia, pancytopenia, and various neuropsychiatric symptoms. However, diagnosing vitamin B12 deficiency lacks a definitive gold standard.

**Case Presentation:** We present two cases where patients initially exhibited pancytopenia with seemingly normal vitamin B12 levels. Based on a bone marrow biopsy, they were initially diagnosed with myelodysplastic syndrome (MDS). Subsequent investigations revealed elevated serum methylmalonic acid (MMA) levels, leading to a revised diagnosis of vitamin B12 deficiency. Both patients showed positive responses to adequate vitamin B12 supplementation.

**Conclusion:** Our case series highlights the importance of ruling out alternative causes of dysplasia in MDS when solely morphological abnormalities are observed on a bone marrow biopsy. It also underscores the crucial aspect of assessing MMA and homocysteine levels in individuals with normal vitamin B12 levels when there is a high clinical suspicion of B12 deficiency.

## 1. Introduction/Background

Vitamin B12 deficiency is often encountered in routine clinical practice. It can present with hematological abnormalities indicative of myelodysplastic syndrome (MDS), a hematological malignancy characterized by pancytopenia, cytogenetic abnormalities, and ineffective hematopoiesis [1]. The diagnosis involves a bone marrow biopsy evaluation and necessitates carefully excluding other potential causes of cytopenia, such as nutritional deficiencies, drug toxicities, infections, and autoimmune diseases [2].

Vitamin B12, or cobalamin deficiency, is a frequently encountered nutritional deficiency, manifesting with a wide range of clinical symptoms [3]. B12 deficiency, although very rarely, can lead to bone marrow failure and dysplasia, resulting in severe pancytopenia without the typical manifestations of this condition [4]. Consequently, if B12 deficiency is not considered in the differential diagnosis of cytopenia's and bone marrow dysplasia, there is a risk of misdiagnosing it as myelodysplastic syndrome. Despite limitations in specificity and ongoing controversies about sensitivity, the measurement of plasma cobalamin remains the gold standard for diagnosing vitamin B12 deficiency. Notably, the results can offer intriguing information in clinical practice but may also be misleading [5].

We present two cases in which patients with vitamin B12 deficiency were initially misdiagnosed as having myelodysplastic syndrome (MDS). Moreover, we demonstrate the potential for grave consequences if nutritional deficiencies are not ruled out, such as unnecessary invasive diagnostic interventions like bone marrow biopsy and treatment with hypomethylating agents [2]. This paper underscores the need for a comprehensive diagnostic assessment of patients with cytopenia and bone marrow dysplasia and aims to shed further light on the need for appropriate diagnosis to ensure appropriate and effective patient care.

## 2. Case Report 1

A 69-year-old female with a history of hypertension visited the Internal Medicine clinic due to bilateral leg weakness accompanied by numbness, tingling, and night sweats. Upon examination, the patient had macrocytic anemia and was referred to the hematology clinic for further investigation. Notably, the patient did not have a history of heavy alcohol consumption.

The laboratory workup revealed a macrocytic anemia, with a hemoglobin level of 9.4 g/dL (a decrease from 12.7 g/dL 2 years ago) and a mean corpuscular volume (MCV) of 131.5 fL. Other blood counts were within normal ranges. Vitamin B12 levels were measured at 1385 pg/mL, and folate levels were normal. Reticulocytopenia was noted, along with normal iron stores. Elevated lactate dehydrogenase (LDH) levels were observed at 546 IU/L, and haptoglobin was undetectable. Direct Coombs test results were negative. Thyroid-stimulating hormone (TSH) and liver function tests (LFTs) were within normal limits. HIV testing yielded negative results. Serum protein electrophoresis with immunofixation did not show any monoclonal protein. The peripheral smear revealed macrocytic normochromic anemia with anisopoikilocytosis, ovalocytes, teardrop forms, and mild neutropenia featuring hypersegmented neutrophils.

Bone marrow biopsy results indicated morphology consistent with myelodysplastic syndrome with single lineage dysplasia (MDS-SLD), mainly affecting more than 10% of megakaryocytes without increased blasts or fibrosis. Notably, no dysplastic changes were reported in the erythroid or granulocytic precursors. Chromosomal analysis revealed a normal female karyotype and fluorescent in situ hybridization (FISH) for the MDS panel with multiple probes was within normal limits.

The initial diagnosis classified the patient with low-risk MDS and assigned a revised international prognostic scoring system (R-IPSS) of 2.5. Her erythropoietin level was measured at 57.86 mU/mL. Treatment was initiated with Epoetin-alfa (Retacrit) at a dosage of 40,000 units weekly. However, due to her anemia's lack of improvement, the dosage was then increased to 60,000 units. Despite the treatment, the patient remained transfusion-dependent, requiring a blood transfusion approximately once every 4–6 weeks.

The patient's leg weakness deteriorated, leading to her becoming wheelchair bound. Subsequent lab tests revealed worsening anemia, progressive thrombocytopenia, and neutropenia. Electromyography (EMG) results indicated a mild mixed axonal and demyelinating peripheral polyneuropathy. A repeat bone marrow biopsy suggested a persistent myeloid neoplasm with features of MDS with multilineage dysplasia, indicative of disease progression. Notably, chromosome analysis and FISH panel remained normal. A comprehensive 51-gene myeloid molecular panel associated with MDS mutations returned negative results.

Given the absence of evidence for a clonal myeloid process beyond multilineage dysplasia, further investigation was undertaken into other causes of bone marrow dysplasia. Upon reevaluation of the peripheral smear, hypersegmented granulocytes were observed despite normal serum B12 levels. Consequently, a serum methylmalonic acid (MMA) level was measured and elevated, suggesting a vitamin B12 deficiency anemia diagnosis.

The patient commenced vitamin B12 injections, improving her symptoms and resolving pancytopenia within the subsequent four months. This outcome underscored the importance of considering nutritional deficiencies, such as vitamin B12, in the differential diagnosis of cytopenia and bone marrow dysplasia.

## 3. Case Report 2

The case involves a 66-year-old female with a history of hypertension who presented to the emergency department with complaints of significant weight loss (40 lbs in one year), fatigue, and a new rash. Laboratory tests revealed pancytopenia, with a white blood cell (WBC) count of 1.8 × 10^9^/L, hemoglobin of 6.5 g/dL (macrocytic), and platelets of 128 × 10^9^/L.

An extensive workup was initiated to investigate the cause of her pancytopenia, ruling out various potential factors such as iron deficiency anemia, vitamin deficiency, gammopathy, infections, heavy metals, and autoimmune causes. Computed tomography (CT) scans of the chest and abdomen/pelvis showed no evidence of occult lymphadenopathy or hepatosplenomegaly. The vitamin B12 level was measured within normal limits at 411 pg/mL. A bone marrow biopsy revealed hypercellular bone marrow (60%–70%) with trilineage hematopoiesis and significant dysplasia in both megakaryocytes and erythroid cells (> 10% in both lines), morphologically indicative of MDS. However, FISH was negative, and the karyotype was normal. Molecular testing was pending due to insurance issues. The International Prognostic Scoring System-Revised (IPSS-R) categorized her as intermediate risk. Given their similar efficacies, she was offered initiation of either IV azacitidine or oral decitabine and opted for oral medications. While her insurance was pending approval for the initiation of her medication, further workup was initiated for other potential causes of her cytopenia. The MMA level was obtained and was greater than 5.00 mmol/L, confirming a true vitamin B12 deficiency. Intrinsic factors were also obtained, and the test was positive. In addition, the patient received a diagnosis of lichen planus based on a skin biopsy of her rash.

B12 deficiency can sometimes mimic MDS, so the patient was initially treated with a 5-day course of Vitamin B12 injections before starting oral decitabine. Subsequently, her blood counts significantly improved with B12 injections, suggesting that B12 deficiency was the primary cause of her pancytopenia rather than MDS. As a result, she was placed on lifelong monthly injections for her B12 deficiency with routine follow-up.

## 4. Discussion

MDS represents clonal myeloid stem cell disorders linked to ineffective hematopoiesis, bone marrow dysplasia, and diminished blood counts [6]. Approximately half of MDS cases exhibit a normal karyotype, and the diagnosis relies on bone marrow biopsy findings demonstrating dysplastic changes in 10% or more of one or more cell lineages [6]. While MDS can be idiopathic, numerous factors, including chemotherapy, radiation, and chemical exposures (such as benzodiazepines and tobacco), can contribute [7]. Before confirming an MDS diagnosis, reversible causes of dysplasia, such as nutritional deficiencies, infections (including HIV), medications, alcohol consumption, and deficiencies in copper, folate, or vitamin B12 must be excluded [7]. A high level of suspicion for MDS is warranted in individuals presenting with cytopenia or symptoms such as infection, bleeding, or fatigue, coupled with dysplastic findings on peripheral smear [7].

The pathogenesis of MDS involves mutations in over 50 recurrently mutated genes, encompassing those encoding proteins involved in pre-mRNA splicing, epigenetic regulation, and transcription [8]. Around 90% of MDS patients harbor at least one oncogenic mutation, with two thirds found in those with a normal karyotype. Treatment primarily focuses on managing symptomatic cytopenias through transfusions and erythropoiesis-stimulating agents [8].

Vitamin B12 deficiency is a frequent cause of megaloblastic anemia and diverse neuropsychiatric symptoms [4]. Initial laboratory assessments should encompass a complete blood count (CBC), peripheral blood smear (for evaluating megaloblastic anemia), and measurement of serum B12 levels [4]. In approximately 50% of individuals with subclinical disease, vitamin B12 levels may appear normal. In such cases, assessing serum MMA and homocysteine levels, which show early elevation in vitamin B12 deficiency, proves to be a more sensitive screening method [9]. Concurrent elevation in MMA and homocysteine levels are 99.8% sensitive for functional B12 deficiency, even when serum B12 levels are within the reference range [9].

In addition to the previously mentioned markers, B12 deficiency can elevate propionyl carnitine (C3), which can indicate conditions such as methylmalonic aciduria and propionic acidemia [10]. This is particularly important in newborns, in whom C3 demonstrates a negative association with maternal B12 levels in the first trimester and, therefore, be a useful biomarker in identifying early vitamin B12 deficiency [10]. In our first case, elevated serum B12 levels were detected, a finding that can signify serious underlying conditions such as liver disease, where cobalamin is released during hepatic cytolysis [11]. Furthermore, elevated B12 has also been observed in hematologic conditions such as promyelocytic leukemia, polycythemia vera, and chronic myelogenous leukemia [11].

The sensitivity and specificity of serum B12 measurements vary primarily due to the absence of a definitive gold standard for diagnosis. Serum B12 levels below 100 pg/mL demonstrate about 90% specificity [12]. However, many diagnostic algorithms set the serum B12 cutoff at 250 pg/mL–300 pg/mL to enhance sensitivity, even though most of these patients may lack hematologic or neurologic manifestations of B12 deficiency [12].

In cases of pernicious anemia, where there are elevated levels of anti-intrinsic factor antibodies, current methods for measuring vitamin B12 levels can yield falsely normal or high results. This is due to the assay's nature, involving binding to intrinsic factor (IF) following dissociation from binding proteins, with a readout based on the remaining unbound IF [13]. If antibodies to IF bind to the test IF reagent, failure of IF-blocking antibodies denaturation can lead to normal or increased measurement of vitamin B12 levels. This phenomenon is mainly observed in conditions like pernicious anemia, where elevated levels of anti-intrinsic factor antibodies are present [13].

In both cases, the patients initially received an MDS diagnosis based on macrocytic anemia and bone marrow biopsy findings. Despite a comprehensive workup, and in our first case, the absence of improvement with transfusions and erythropoiesis-stimulating agents, along with unexpectedly normal FISH/cytogenetics, prompted a reconsideration of the MDS diagnosis. Therefore, alternative causes of macrocytic anemia in both cases were explored, leading to the diagnosis of vitamin B12 deficiency.

## 5. Conclusion

This case series underscores the critical importance of thoroughly investigating alternative causes of dysplasia in MDS cases where only morphological abnormalities are evident on bone marrow biopsy. Failure to explore secondary causes of cytopenia through bone marrow biopsy could result in a misdiagnosis of MDS, causing unwarranted emotional distress for the patient and inappropriate administration of hypomethylating agents. In addition, it highlights the necessity of assessing MMA and homocysteine levels in patients exhibiting normal vitamin B12 levels but with a heightened clinical suspicion of B12 deficiency. Given the lack of definitive diagnostic criteria for vitamin B12 deficiency, we advocate for comprehensive screening protocols, particularly in cases with significant suspicion, incorporating evaluations of MMA and homocysteine levels. Furthermore, in instances of pernicious anemia, where B12 levels may be falsely elevated due to the presence of anti-intrinsic factor antibodies, assessing both MMA and anti-intrinsic factor antibodies can aid in establishing an accurate diagnosis.

## Data Availability

The clinical and pathologic data used to support the findings of this study are included within the article.
